# Incidental Intestinal Schwannoma in a Patient of Ulcerative Colitis With Adhesive Intestinal Obstruction: A Case Report

**DOI:** 10.7759/cureus.22343

**Published:** 2022-02-17

**Authors:** Archana Khanduri, Naga Bharati Musthalaya, Arvind Singh, Jyoti Gupta, Rahul Gupta

**Affiliations:** 1 Gastrointestinal Surgery, Synergy Institute of Medical Sciences, Dehradun, IND; 2 Histopathology, American Institute of Pathology and Laboratory Sciences, Citizens Hospital, Hyderabad, IND; 3 Gastroenterology, Synergy Institute of Medical Sciences, Dehradun, IND; 4 Radiation Oncology, Himalayan Institute of Medical Sciences, Dehradun, IND

**Keywords:** bowel resection, laparotomy, ileum, intestinal obstruction, ulcerative colitis, schwannoma

## Abstract

Gastrointestinal (GI) schwannomas are very rare. Among GI schwannoma, the most common site is the stomach. Very few cases of intestinal schwannoma have been reported in the literature. Moreover, intestinal schwannoma in the setting of inflammatory bowel disease has rarely been reported. We report a case of ileal schwannoma incidentally detected in a 52-year-old lady with long-lasting ulcerative colitis having intestinal obstruction. On laparotomy, the patient was found to have adhesive intestinal obstruction due to a previous abdominal hysterectomy. On careful inspection of the bowel loops, a 2cm ileal lesion on the mesenteric border was detected. Segmental ileal resection was performed. Histopathology revealed a mesenchymal tumor with immunohistochemical analysis suggestive of schwannoma (CD117-, CD34-, SMA-, and S100+). The patient had an uneventful recovery with no recurrence on follow-up.

## Introduction

Schwannoma is a type of peripheral nerve sheath tumor. It is most commonly seen in the head, neck, and limbs. It is a rare mesenchymal tumor of the gastrointestinal (GI) tract. It accounts for 2.9% of all surgically removed mesenchymal tumors of the GI tract [1]. Most of them are incidentally detected during screening endoscopy, colonoscopy, or radiological imaging [2-4]. Larger lesions give rise to symptoms such as abdominal pain, vomiting, melena, and abdominal distension similar to that observed in patients with other GI tumors [5-7]. We report a case of small intestinal schwannoma incidentally detected during surgery in a patient suffering from ulcerative colitis (UC), who was operated for adhesive intestinal obstruction.

## Case presentation

**Figure 1:**
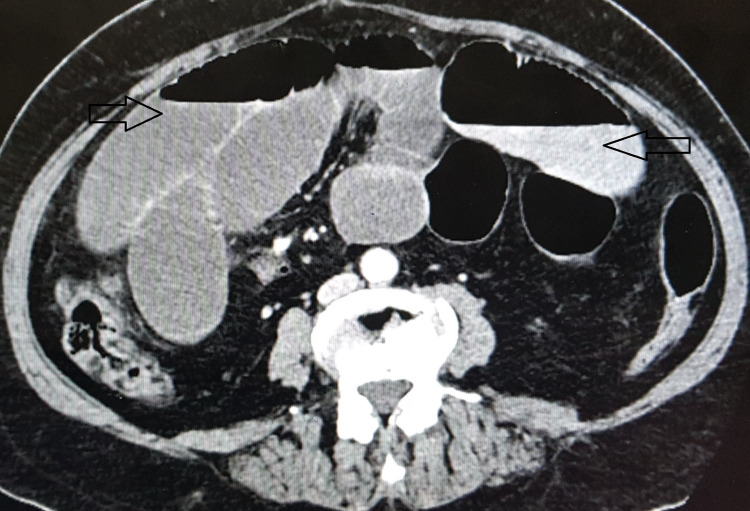
Computed tomography of the abdomen showing dilated small bowel loops with air-fluid levels (arrows).

A 52-year-old lady presented with abdominal pain, bilious vomiting, constipation, and abdominal distension for two days. She was diagnosed to have moderately active UC involving the whole colon (pancolitis) four months back and was treated with oral mesalamine therapy. She had undergone abdominal hysterectomy for uterine fibroids eight years back. On clinical examination, the patient was hemodynamically stable. Abdomen was distended with hyperperistaltic bowel sounds. Routine blood investigations were within normal limits. Computed tomography (CT) of the abdomen revealed dilated proximal ileal loops with air-fluid levels and collapsed terminal ileal loops (Figure 1). There was abrupt narrowing and ‘bird beak appearance’ in the proximal ileum located underneath the anterior abdominal wall. In view of above findings, the patient was planned for emergency laparotomy. 

**Figure 2:**
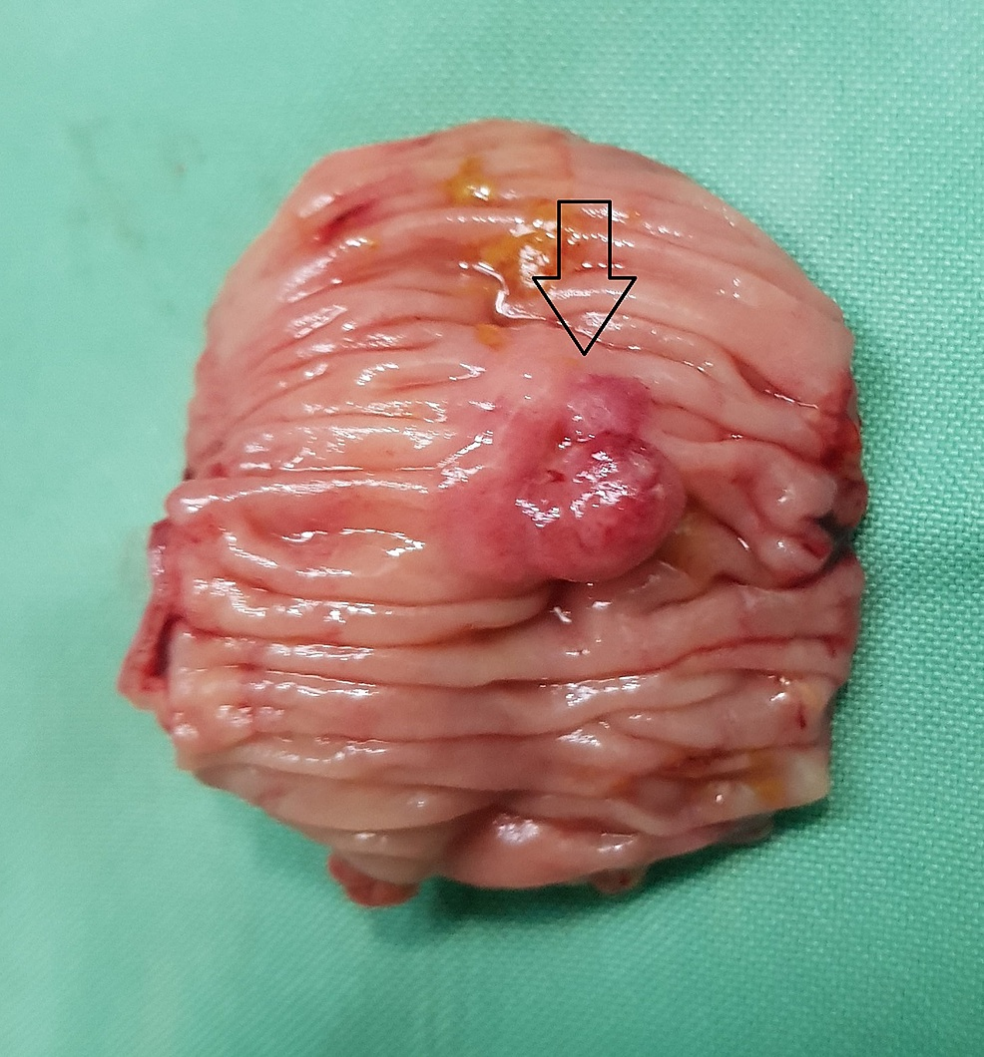
Mucosal surface of the resected small bowel showing the nodular lesion with central umbilication (arrow).

On laparotomy, the proximal ileal loops were grossly dilated and the terminal ileal loops were collapsed. The large bowel loops were also collapsed and appeared unremarkable. The ileal loops were densely adhered to the anterior abdominal wall at the previous scar site causing luminal obstruction. Extensive adhesiolysis was done and the obstruction was released. On careful examination, a 2 x 2 cm lesion was noted on the mesenteric side of the terminal ileum distal to the site of adhesive obstruction (Figure 2). In view of the suspicion of small bowel tumor, the involved segment of the ileum was resected and end-to-end ileo-ileal anastomosis was performed. The postoperative course was uneventful with the hospital stay of four days. 

**Figure 3:**
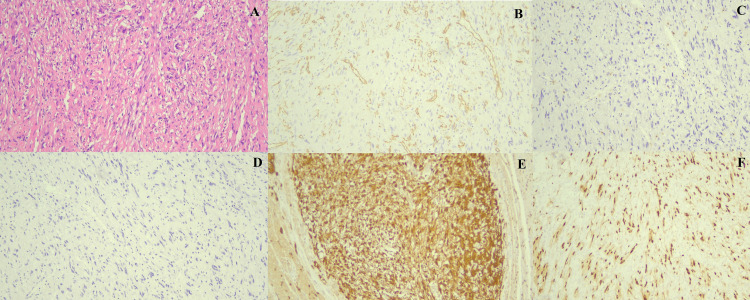
Microscopic examination of the resected specimen revealed elongated spindle shaped cells having ovoid hyperchromatic nuclei and eosinophilic cytoplasm with fascicular and focal haphazard arrangement (A). Immunohistochemistry showed negative staining for CD34 (B), CD117 (C), and DOG1 (D), and positive staining with S100 (E) and SOX10 (F).

Histopathology revealed ulcerated polypoidal lesion with spindle cells arranged in fascicles. The spindle cells had moderate amount of eosinophilic cytoplasm and ovoid hyperchromatic nuclei (Figure 3). On immunohistochemistry, the tumor cells showed positive staining for S100, ki67, and SOX10 and negative staining for CD34, CD117, DOG-1, and SMA (Figure 3). Based on these findings, the final pathological diagnosis of intestinal schwannoma was made. On the last follow-up at two months after surgery, the patient was symptom free and receiving oral mesalamine as the maintenance therapy for UC.

## Discussion

Mesenchymal tumors of GI tract are relatively uncommon compared to other neoplasms. Among them, gastrointestinal stromal tumor (GIST) is the most common tumor [8]. In a large series of 3250 GI mesenchymal tumors, only two cases of duodenal schwannomas were reported [9]. The stomach is the most common site of GI schwannoma [1]. In one of the largest series of 33 digestive schwannomas, none of them were located in the small intestine [1]. There are anecdotal reports of small intestinal schwannoma, as seen in the present case [5-7,10]. In some case series, both sexes are equally affected [1], while in other case series, females are more commonly affected [11]. Most patients are in the age group of 40-60 years with a median age of 52 years [1,11]. The reported tumor size ranges from 1 cm to 12 cm [1]. 

Preoperative diagnosis of GI schwannoma is difficult. Small lesions are often not visible on radiological investigations such as CT and magnetic resonance imaging (MRI) as seen in the present case. On CT abdomen, the schwannomas appear as exophytic or endophytic, round to oval lesions with heterogenous enhancement [11]. Larger lesions may show central necrosis, calcification, and surface ulceration. However, none of these CT features are unique to schwannomas and are observed in other mesenchymal tumors such as gastrointestinal stromal tumor (GIST) [10]. On upper GI endoscopy or colonoscopy, the tumors are visible as submucosal lesions. A biopsy can help in making the diagnosis of schwannoma. Most patients require endoscopic or surgical excision due to diagnostic dilemma or to relieve the symptoms. Small lesions (< 2cm) located in the stomach, colon, or rectum can be excised endoscopically. However, larger lesions and those located in the small intestine often require surgical resection. No recurrence after complete surgical resection has been reported in the follow-up studies [1].

On histology, all mesenchymal tumors are typically composed of spindle cells. Immunohistochemistry is required to determine the origin of the tumor. Schwannomas are characterized by immunopositivity for S100, vimentin, and negative staining for CD34, CD117, actin, and desmin, as seen in the present case [1]. The malignant potential of mesenchymal tumors is estimated based on mitotic rate, Ki67 index, presence of atypical features, tumor size, and the extent of tumor necrosis [8].

There is an increased risk of colorectal cancer and small bowel adenocarcinoma in patients with inflammatory bowel disease [12,13]. However, there are very few reports of GI non-GIST mesenchymal tumors in these patients [4,14-15]. So, we believe that the occurrence of GI schwannoma in inflammatory bowel disease seems coincidental. Future larger studies are required to determine the actual risk of GI mesenchymal tumors in patients with inflammatory bowel disease.

## Conclusions

Schwannoma should be included in the differential diagnosis of small bowel tumors. Preoperative diagnosis of intestinal schwannoma is difficult. Surgical resection is the mainstay of treatment. It is a benign tumor and associated with a good prognosis. It should be differentiated from GIST on histology using immunohistochemical analysis.
